# The Immune Microenvironment of Chordomas: An Immunohistochemical Analysis

**DOI:** 10.3390/cancers13133335

**Published:** 2021-07-02

**Authors:** Maroa Dridi, Lila Krebs-Drouot, David Meyronet, Jean Marc Dumollard, François Vassal, Emmanuel Jouanneau, Timothée Jacquesson, Cédric Barrey, Sylvain Grange, Jean Boutonnat, Michel Péoc’h, Georgia Karpathiou

**Affiliations:** 1 Pathology Department, University Hospital of Saint-Etienne, 42055 Saint-Etienne, France; maroa.dridi@etu.univ-st-etienne.fr (M.D.); j.marc.dumollard@chu-st-etienne.fr (J.M.D.); michel.peoch@chu-st-etienne.fr (M.P.); 2 Pathology Department, University Hospital of Grenoble, 38700 Grenoble, France; lkrebsdrouot@chu-grenoble.fr (L.K.-D.); jboutonnat@chu-grenoble.fr (J.B.); 3 East Pathology Institute, Hospices Civils de Lyon, 69677 Lyon, France; david.meyronet@chu-lyon.fr; 4 Cancer Cell Plasticity Department, Cancer Research Center of Lyon, 69373 Lyon, France; 5 Claude Bernard University, Lyon 1, 69100 Lyon, France; emmanuel.jouanneau@chu-lyon.fr (E.J.); c.barrey@wanadoo.fr (C.B.); 6 Neurosurgery Department, University Hospital of Saint-Etienne, 42055 Saint-Etienne, France; francois.vassal@chu-st-etienne.fr; 7 Department of Neurosurgery B, Neurological Hospital Pierre Wertheimer, 69500 Lyon, France; timothee.jacquesson@neurochirurgie.fr; 8 Inserm U1052, CNRS UMR5286, «Signaling, Metabolism and Tumor Progression» The Cancer Research Center of Lyon, 69373 Lyon, France; 9 Department of Anatomy, Faculté de Médecine Lyon-Est, Université de Lyon, Université Claude Bernard Lyon 1, 69100 Lyon, France; 10 Department of Spine and Spinal Cord Surgery, Neurological Hospital Pierre Wertheimer, 69500 Lyon, France; 11 Radiology Department, University Hospital of Saint-Etienne, 42055 Saint-Etienne, France; sylvain.grange@chu-st-etienne.fr

**Keywords:** PD-L1, CD8, CD4, CD20, macrophages, MECA-79, vascular density, immunohistochemistry, prognosis

## Abstract

**Simple Summary:**

Chordoma patients may be amenable to immunotherapy; however, the immune microenvironment of chordomas needs further investigation. We performed the immunohistochemical analysis of a chordoma series, showing that these tumors have a unique microenvironment characterized by the absence of PD-L1 tumor cell expression, but feature PD-L1+ immune cells playing a negative prognostic role.

**Abstract:**

Chordomas are rare sarcomas that are usually treated by surgery and/or radiotherapy since these are chemo-resistant tumors, but immunotherapy could be a possible option for chordoma patients. However, few reports investigating the composition of the chordoma immune microenvironment exist. We immunohistochemically studied 81 chordomas regarding their immune microenvironment factors and compared them with clinicopathological data. Macrophages and CD4 cells were the most prominent inflammatory cell populations, followed by CD8 T cells, while CD20 B cells and high endothelial venules (MECA-79+) were less frequently found. PD-L1 (22C3) expression by inflammatory cells was found in 21 (26%) tumors and was associated with a larger tumor size. None of the cases showed the expression of PD-L1 by tumor cells. Survival analysis showed that younger patients had a better overall survival. Considering the immunohistochemical factors studied, higher CD8, the presence of PD-L1+ immune cells, and higher vascular density were adverse prognostic factors, but in multivariate analysis, only PD-L1+ immune cells retained prognostic significance. To conclude, chordoma tumor cells do not express PD-L1, but PD-L1+ immune cells seem to play a negative prognostic role, supporting the need for further studies in this field and the possible beneficial role of immunotherapy in these patients.

## 1. Introduction

The tumor immune microenvironment has become a major subject of recent literature, revealing important pathophysiological pathways in various tumors [1,2]. Treatments intervening in the tumor microenvironment with the aim to boost the cytotoxic effects of patients’ immune responses have gained great interest after showing efficacy in numerous malignancies [3]. Fewer data exist regarding the details of the immune microenvironment of sarcomas [4]. Sarcomas are generally considered immune-quiescent tumors since they have a low mutational burden, but recent data suggest that some sarcomas are immunogenic [4]. Chordomas are rare sarcomas that usually affect the axial skeleton—the skull base, the sacrum, and the spine—and are believed to be derived from notochordal remnants [5]. Surgical resection and radiotherapy are cornerstone treatments in this area since these are chemo-resistant tumors [5]. However, immunotherapy could be a possible option for chordoma patients, as for patients with other types of sarcomas [6], with some promising results being shown in the rare cases reported [7]. Currently, a phase I trial of nivolumab with or without stereotactic radiosurgery in chordoma patients is taking place (ClinicalTrials.gov Identifier: NCT02989636). However, few reports investigating the composition of the chordoma immune microenvironment exist.

To the best of our knowledge, one of the first reports on the interaction between chordomas and the host immune system was conducted in chordoma cell lines and chordoma tissues from 10 patients, showing PD-L1 and PD-1 expression in some immune cells of the chordoma tissues but no PD-L1 expression in tumor cells [8]. Another study on chordoma cell lines and tumor microarrays published almost at the same time showed PD-L1 expression in more than 90% of the cases studied [9]. The same study evaluated the presence of immune cells on morphological grounds and found a statistical trend of tumors being highly infiltrated by immune cells and showing a worse prognosis. Later, an immunohistochemical and immunofluorescence study of 54 chordoma samples for PD-1, PD-L1, CD3, CD8, CD4, CD20, and FOXP3 expression showed PD-L1 tumor cell expression in almost 70% of the cases and suggested that the higher infiltration of CD3, CD8, and CD4 T cells is a positive prognostic factor [10,11,12,13,14].

Thus, given the rarity of these tumors and the limited and often contradictory results in the literature, we aimed to investigate a chordoma series for the expression of immune microenvironment factors and compare it with the clinicopathological characteristics.

## 2. Materials and Methods

In this multicenter retrospective cohort study, we included 91 patients with a histologically confirmed chordoma diagnosis between 1998 and 2020. The Local Ethics Committee approved the study (IRBN702020/CHUSTE). Diagnosis was based on clinicoradiological data, typical morphological features, and S100/cytokeratin expression, and it was centrally reviewed by a specialized soft-tissue pathologist (Michel Péoc’h).

Only conventional chordomas, and not dedifferentiated or poorly differentiated subtypes, were included for homogeneity reasons. Follow-up data were available for 85 patients, and tissue was not sufficient for another 4 patients; thus, tissues from 81 patients were further studied by immunohistochemistry. The age of the archival tissue and its decalcification were recorded to assure quality issues.

Whole-tumor sections were studied by immunohistochemistry for PD-L1 (22C3, Dako Agilent, Santa Clara, CA, USA 1/40), CD8 (C8/144B, Dako Agilent, Santa Clara, CA, USA 1/100), CD4 (SP35, Abcam, Cambridge, UK 1/50), CD20 (L26, Dako Agilent, Santa Clara, CA, USA 1/200), CD163 (10D6, Novocastra, Newcastle upon Tyne, UK 1/200), CD34 (QBEnd10, Dako Agilent, Santa Clara, CA, USA 1/800), and MECA-79 (MECA-79, Santa Cruz Biotechnology, Dallas, TX, USA, 1/750) (a high endothelial venule marker associated with tertiary lymphoid structures [4]) expression. It was performed in one of our departments using an automated staining system (OMNIS, Dako-Agilent, Santa Clara, CA, USA) and the EnVision FLEX kit (OMNIS, Dako, Denmark) according to the manufacturer’s protocol. Positive immunoreactions were visualized using 3,3′-diaminobenzidine as the chromogenic substrate and HRP magenta (Dako, Agilent) as the second chromogen for double immunostains (PD-L1 with CD8 and PD-L1 with CD163).

Immunohistochemical evaluation for CD8, CD4, CD20, CD163, and PD-L1 expression by immune cells was performed, as previously described, in a semiquantitative manner: 0, no cells; 1, few cells (<10%); 2, a moderate number of positive cells (≥10% and <40%); and 3, abundant cells (≥40%). A binary system of low (scores 0 and 1) and high (scores 2 and 3) scores for CD8, CD4, CD20, and CD163 was used for further statistical analysis [3,11], while a system of absent (score 0) or present (score 1–3) was used for PD-L1+ immune cells. Immune cells were predominantly found in the peritumoral compartment, and assessment was performed for this immune cell compartment. The quantification of the number of CD34+ and MECA-79+ blood vessels (mean vessel density, hereafter called vascular density) was performed on 5 high-power 20× (1 mm^2^) fields per section that were counted and averaged, as previously proposed [15]. They were further classified into two groups using the median as the cut off value.

Data were analyzed using the StatView software version 5 (Abacus Concepts, Berkley, CA, USA). We used the χ^2^ test (confirmed by Fisher’s exact test) to explore any relationship between two groups for categorical data. Survival probability was estimated by Kaplan–Meier analysis with log-rank product limit estimation. For all analyses, statistical significance was indicated at a *p* value of <0.05.

## 3. Results

The patients’ characteristics are shown in Table 1. The mean age at diagnosis was 58 years, with a median of 64 years, which was used as a cut off value for further analysis. A total of 47 (58%) patients were male, while 34 (42%) were female. The skull was the most frequent localization (*n* = 30, 37%), followed by the sacrum and the mobile spine. Median tumor size (available for 38 cases) was 43 mm (19–144 mm), and this was used as a cut off value for further comparisons. Surgical treatment was most often used (*n* = 74, 84.7%), followed by adjuvant therapy in almost half of the cases. Follow up ranged from 2 to 264 months (median 60, mean 69.5 ± 59.5). Recurrences were noted in 52 patients (64.2%), and there were multiple recurrences in 24 (46.2%). Fifteen patients (18.5%) died due to the disease. The median overall survival (log-rank) was not reached.

Immunohistochemical data are shown in Table 2. Results were available for 81 tumors for PD-L1 and CD8, 74 tumors for CD163 and CD34, 73 tumors for CD20, 41 tumors for CD4, and 59 tumors for MECA-79. Further statistical analysis was performed only for these cases.

**Figure 1:**
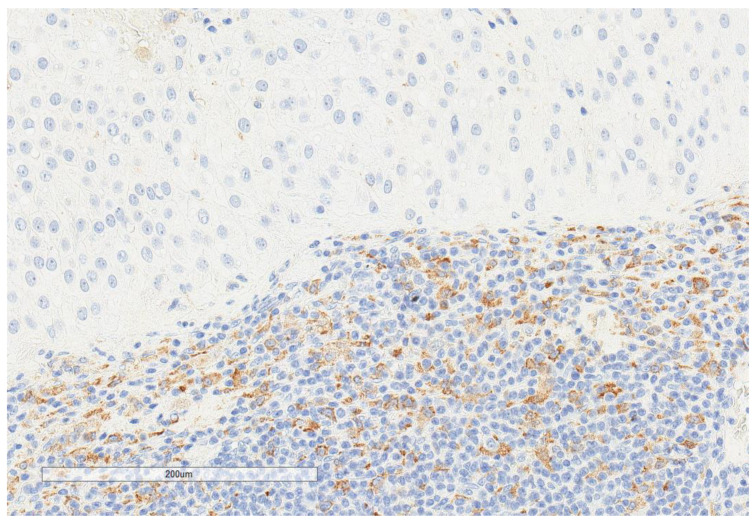
Representative microscopic image (×200 magnification) of high CD163 expression. The upper part of the image shows the tumor, and the lower part shows the inflammatory cells.

**Figure 2:**
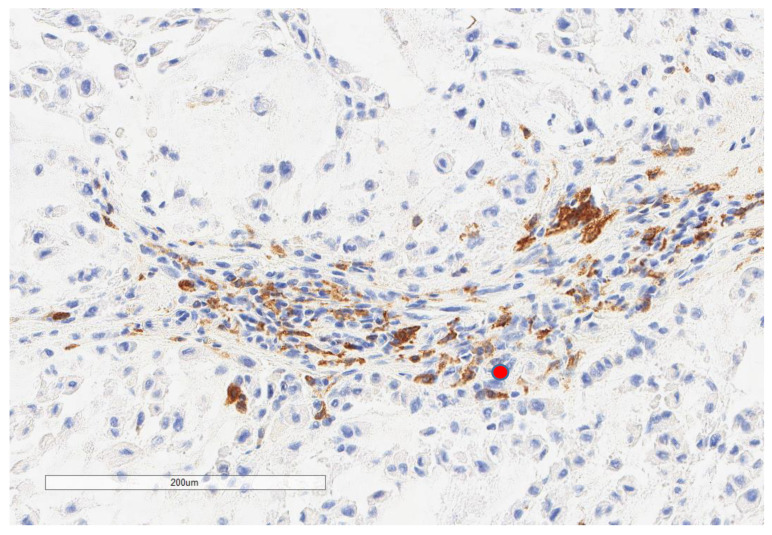
Representative microscopic image (×200 magnification) of low CD163 expression (red circle).

**Figure 3:**
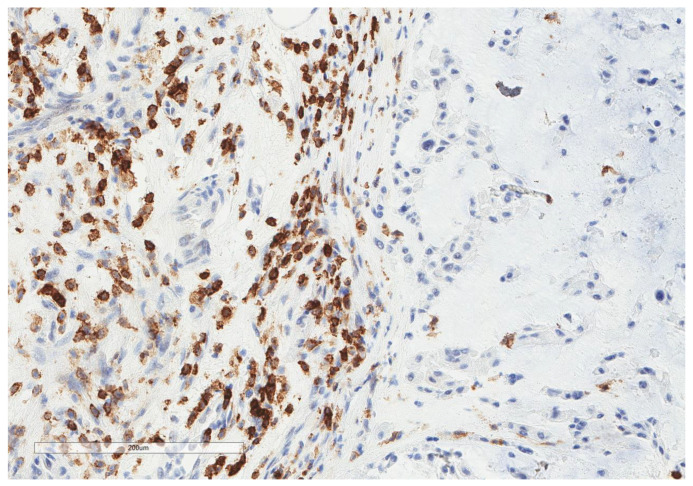
Representative microscopic image (×200 magnification) of high CD4 expression. The right part of the image shows the tumor, and the left part shows the inflammatory cells.

**Figure 4:**
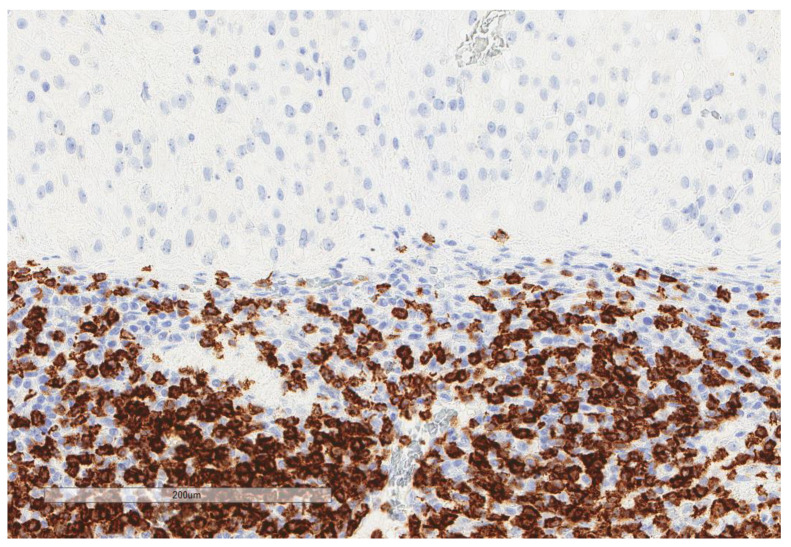
Representative microscopic image (×200 magnification) of high CD8 expression. The upper part of the image shows the tumor, and the lower part shows the inflammatory cells.

**Figure 5:**
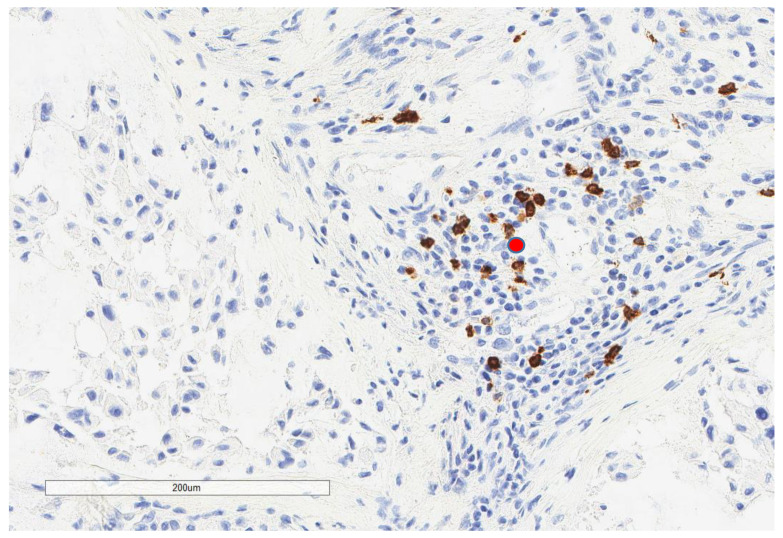
Representative microscopic image (×200 magnification) of low CD8 expression (red circle).

**Figure 6:**
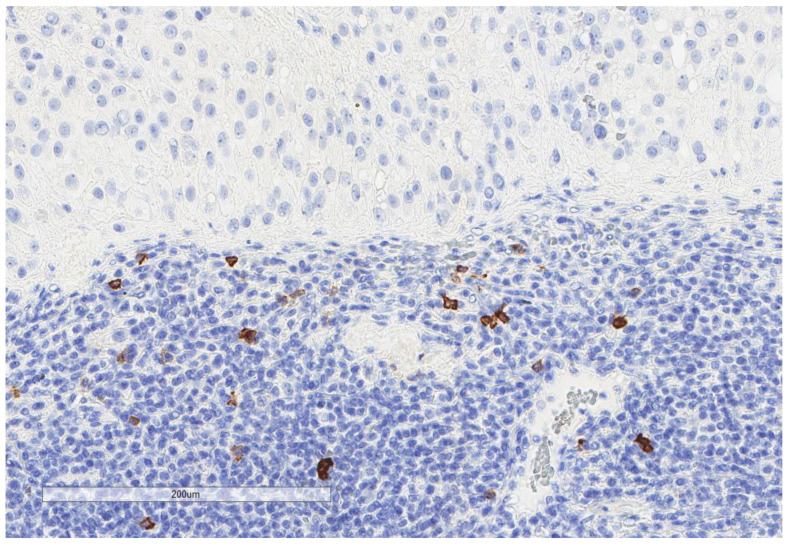
Representative microscopic image (×200 magnification) of low CD20 expression. The upper part of the image shows the tumor, and the lower part shows the inflammatory cells.

**Figure 7:**
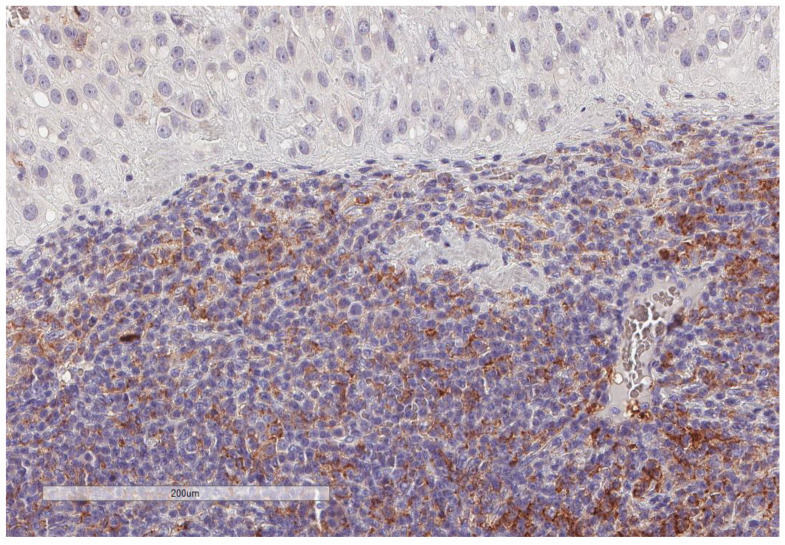
Representative microscopic image (×200 magnification) of PD-L1 expression but not tumor cells. The upper part of the image shows the tumor, and the lower part shows the inflammatory cells.

**Figure 8:**
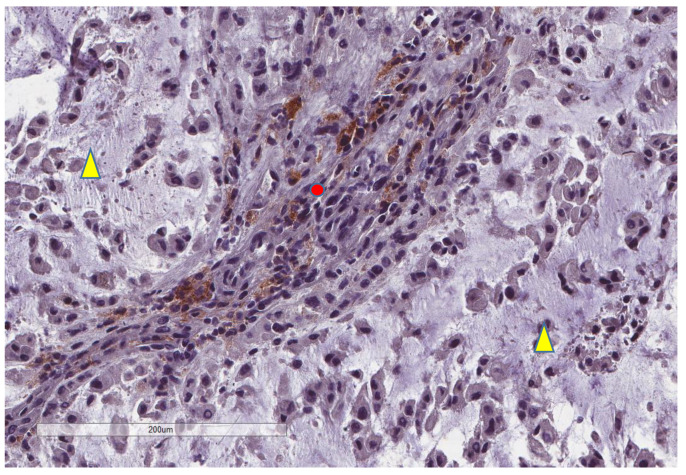
Representative microscopic image (×200 magnification) showing PD-L1 expression by immune cells (red circle) but not tumor cells (yellow triangle).

**Figure 9:**
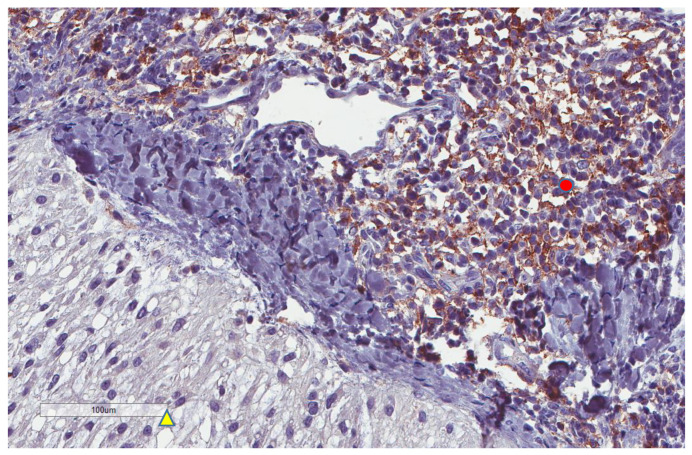
Representative image (×200 magnification) of another case showing that tumor cells (yellow triangle) do not express PD-L1, whereas nearby immune cells are PD-L1+ (red circle).

**Figure 10:**
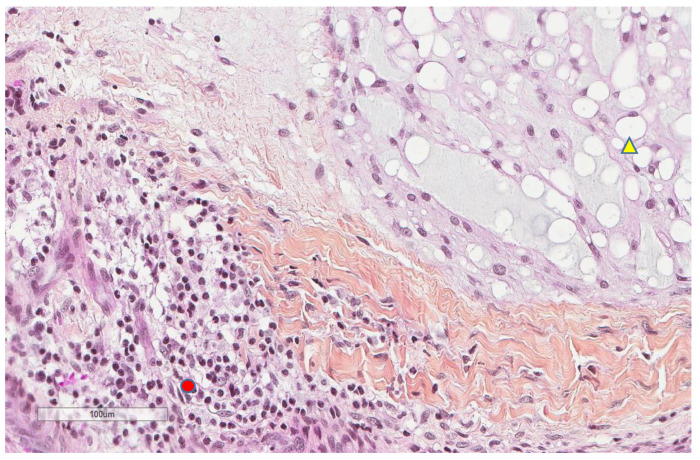
The morphologic features of the same focus previously shown (×200): immune cells (red circle) and tumor cells (yellow triangle).

**Figure 11:**
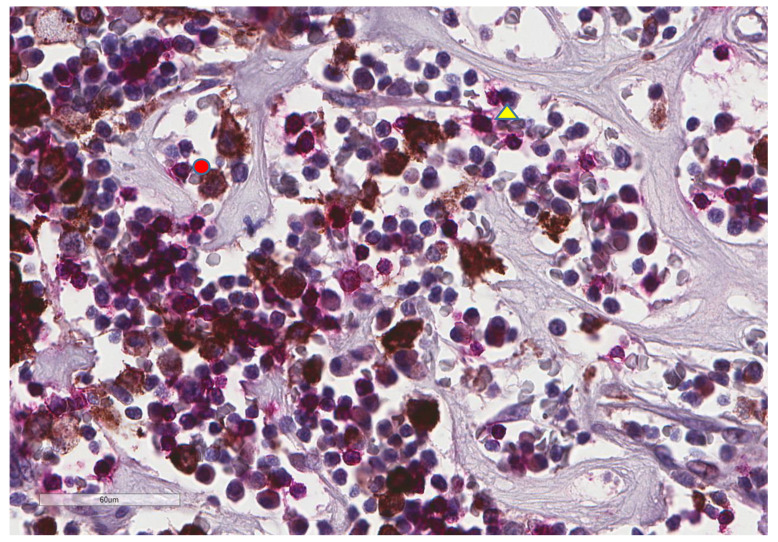
Representative microscopic image of a double stain for PD-L1 (DAB = brown) and CD8 (magenta), showing that most CD8 cells (yellow triangle) do not express PD-L1, which is found in larger cells (red circle) (×400 magnification).

**Figure 12:**
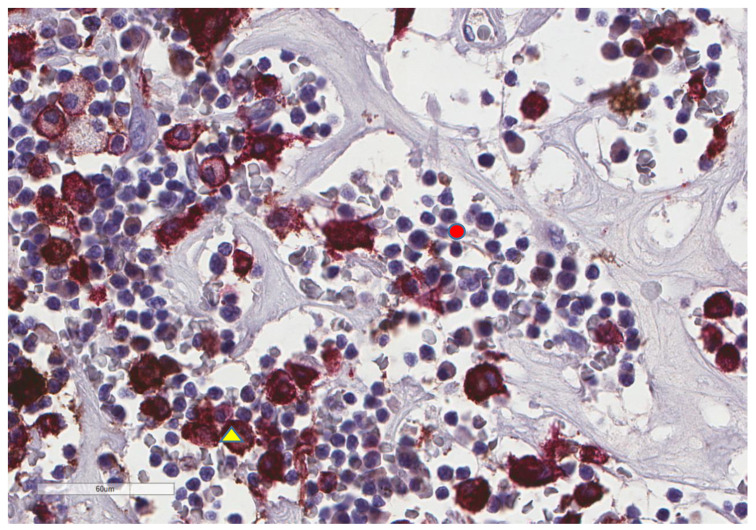
Representative microscopic image of a double stain for PD-L1 (DAB = brown) and CD163 (magenta), showing that most CD163 cells also express PD-L1 (yellow triangle), while the background lymphocytes (red circle) are negative (×400 magnification, same focus as in the previous figure).

**Figure 13:**
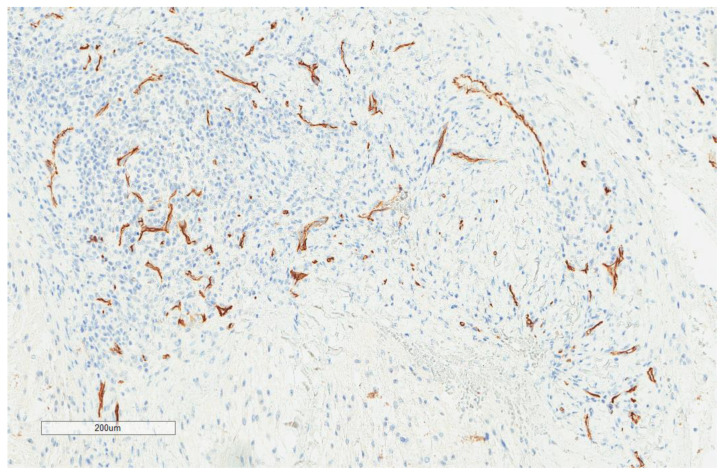
Representative image of CD34 vascular density (×100).

**Figure 14:**
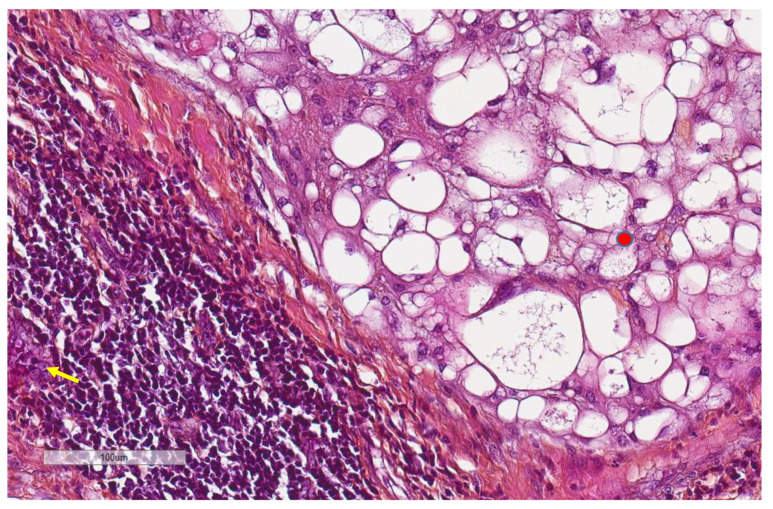
Representative image (×200 magnification) of the morphological aspect of high endothelial venules (arrow). Nearby, the tumor (red circle) cells are shown.

**Figure 15:**
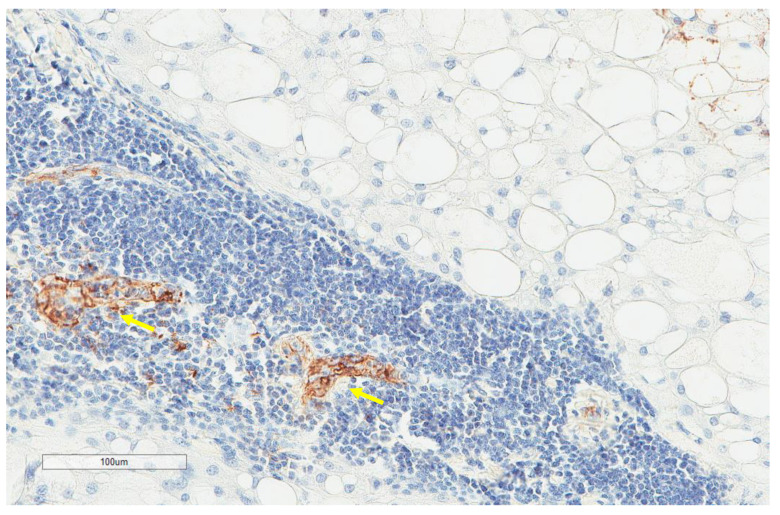
Representative images (same tumor focus as previous image at ×200 magnification) with the corresponding MECA-79+ high endothelial venules (yellow arrows).

**Figure 16:**
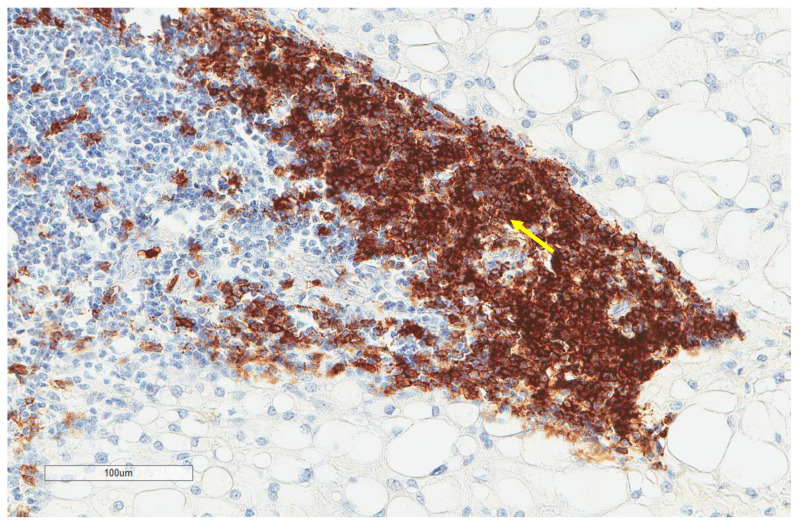
Representative image (same tumor focus as previous image at ×200 magnification) showing CD20+ B cells (yellow arrows).

CD163+ macrophages and CD4+ T cells (Figure 1, Figure 2 and Figure 3) were the most prominent inflammatory cell population, with 39.2% tumors showing high CD163+ and 39% showing high CD4+ infiltration; followed by CD8 cytotoxic T cells (Figure 4 and Figure 5), with 26 (30.5%) tumors showing high CD8 infiltration; while CD20 B cells (Figure 6 and Figure 16) were less frequently found, with 8 (11%) tumors showing high infiltration. PD-L1 expression by inflammatory cells (Figure 7, Figure 8, Figure 9, Figure 10, Figure 11 and Figure 12) was found in 21 (26%) tumors. The morphology of these PD-L1+ immune cells, as well as the double immunostaining of PD-L1 with CD8 and CD163, suggested that these were predominantly macrophages (Figure 11 and Figure 12). None of the cases showed PD-L1 expression by tumor cells. Vascular density (Figure 13) varied from 1 to 22 vessels per field (median 3.5), and 37 (50%) tumors showed a high vascular density. MECA-79+ vessels (Figure 14 and Figure 15) were found in only 5 (8.5%) tumors, with a density of 1 to 4 vessels per field, and associated with perivascular lymphoid structures (Figure 14, Figure 15 and Figure 16).

A comparison between the immunohistochemical factors studied (Table 3, χ^2^ test confirmed by Fisher’s exact test) showed a positive correlation between CD8 and CD20 cells (*p* = 0.01) and a strong trend (*p* = 0.05) for positive CD8 and CD163 correlation. The presence of PD-L1-positive immune cells was associated with a higher presence of CD8 (*p* = 0.0007) and CD163 (*p* = 0.0004) cells, as well as a higher vascular density (*p* = 0.0005). Vascular density was also associated with higher CD8 (*p* < 0.0001) and CD163 (*p* = 0.03) densities. CD4 infiltration was not associated with CD8 (*p* = 0.09), CD163 (*p* = 0.08), or CD20 (*p* = 0.1); it was associated with PD-L1 (*p* = 0.01, 66.7% of tumors with PD-L1 immune cell expression showed high CD4 infiltration vs. 33.3% showed a low CD4 infiltration). PD-L1 expression by immune cells was also associated with larger tumors (*p* = 0.003). Localization, sex, and age were not associated with any of the factors studied.

In order to assess the possible impact of tissue condition given the retrospective nature of the study, we compared the immunohistochemical findings with the age of the archival tissue using 5 years (as indicated by the PD-L1 22C3 clone manufacturer Dako Agilent) as the cut off value (51 tumors >5 years, 30 tumors ≤5 years, median 7, range 1–22, mean 7.8 years), as well as with its decalcification (22 tumors were decalcified: ethylenediaminetetraacetic acid (EDTA) for 13 cases, formic acid for 5, RDO for 3 cases, and nitric acid for 1). We found that older tissues had a lower CD8 expression (21% for old tissue and 70.8% for young tissue, *p* = 0.0003) and PD-L1 expression (13.1% for old tissue and 54.1% for young tissue, *p* = 0.001) in comparison to more recent tissue samples, without differences for the rest of the factors studied. Decalcification did not seem to impact the factors studied (PD-L1 expression (*p* = 0.3), CD8 expression (*p* = 0.1)), but given the different agents used this should be interpreted with caution. To investigate if the tissue age had an impact on the survival analysis, data were also stratified according to the 5-year cut off value of tissue age, showing that statistical significance was retained and was not associated with the tissue age.

**Figure 17:**
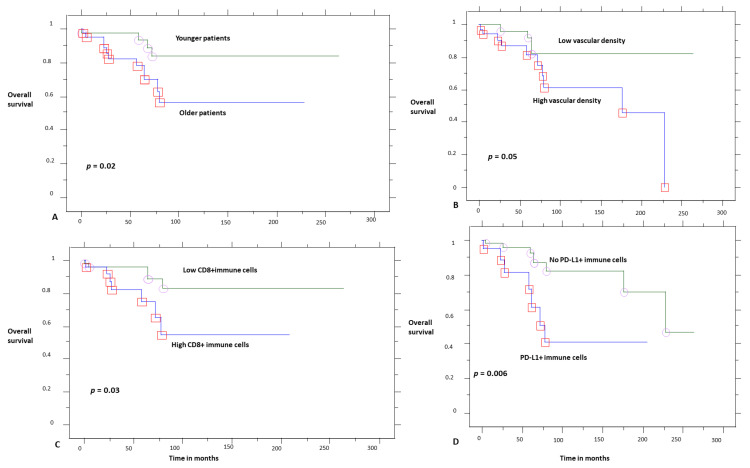
Overall survival according to age (**A**), vascular density (**B**), CD8 (**C**), and PD-L1 expression by immune cells (**D**). Circles and red squares correpsond to event times.

The survival analysis (Table 4, Kaplan–Meier analysis) showed that younger patients (Figure 17) had a better overall survival (10-year OS 82% vs. 58%, *p* = 0.02). Considering the immunohistochemical factors studied (Figure 17), higher CD8 infiltration (10-year OS 81% vs. 51%, *p* = 0.03), the presence of PD-L1+ immune cells (10-year OS 81% vs. 46%, *p* = 0.02), and higher vascular density (marginally, *p* = 0.05, 10-year OS 81% vs. 60%) were adverse prognostic factors.

Further stratification (Kaplan–Meier analysis) of the OS for PD-L1 and CD8 expression showed that tumors infiltrated by CD8+ and PD-L1+ immune cells had a worse prognosis (10-year OS 18%), while tumors with a high CD8 expression but without PD-L1 immune cell expression (10-year OS 91%) had better survival (*p* = 0.05). Furthermore, Cox multivariate regression analysis (Table 5) for the significant factors of the univariate analysis showed that only PD-L1 retained prognostic significance, while age and CD8 expression were not statistically significant factors.

## 4. Discussion

The current series is one of the largest in chordomas, investigating their immune microenvironment. We show that PD-L1 is not expressed by tumor cells in these tumors, which is a striking difference from two previous studies: Feng et al. reported PD-L1 tumor cell expression in 94.9% of their cases, as studied in tissue microarrays [9], while Zou et al. found PD-L1 expression by tumor cells in 68.5% of 54 cases studied, as reported in four different studies [10,11,13,14], and 66.7% in their validation series of 60 patients [12]. On the contrary, our results confirm the observations of Mathios et al. who reported no PD-L1 expression by tumor cells in 10 samples studied [8].

We believe that the most plausible explanation for these discrepancies is the different immunohistochemical techniques used, especially for PD-L1, which is known to vary even between well-established clones [16]. We used an antibody (22C3) that is often used in many clinical trials and in routine practice for theranostic reasons. Mathios et al. used a 5H1 clone [8], while Feng et al. did not report a reference for the antibody used [9]. Zou et al. used a monoclonal antibody with the reference ab174838 in the first report [13], while in the three next reports of the same samples they referred to the clone 28-8 [10,11,14] (ab205921, which replaces the ab174838 clone, which was discontinued as denoted in the company site); in their last report of the same cohort and the validation cohort, they referred to their previous works for protocol details [12], so this is probably the 28-8 clone. These discrepancies between the various studies highlight the difficulties in immunohistochemical techniques and justify the need for more studies in different patient populations and using different techniques. We also considered possible the tissue age, and thus its antigenicity, as a parameter of variability, given the retrospective nature of the study, despite the fact that the previous studies were also retrospective and, when mentioned, the inclusion time was also large in these studies (2002–2015 for the 54 samples of Zou et al. [10,11,13,14] and 2006–2018 for their validation cohort [12]). This question of tissue quality has not been posed in the previous works. We did find a diminution of staining with time, but this did not impact the prognosis since statistical significance was retained after stratifying for this parameter. The same observation of diminution in PD-L1 expression has been also made in a large series of lung cancer samples tested for PD-L1 expression, but it was considered a possible consequence of previous treatments and not of time itself [17]. Here, in an untreated cohort, we show that time indeed impacts this expression, and this highlights the need to perform these techniques shortly after diagnosis, especially for diseases that are not expected to recur soon after initial treatment, such as chordomas.

The prognostic impact that we found here refers to PD-L1-positive immune cells, notably macrophages, which act as a negative prognostic marker. This is pathophysiologically explained by the inhibitory role that they play in the tumor microenvironment since PD-L1, which is expressed in tumor cells and/or immune cells, interacts with the PD-1 expressed on activated T cells, causing their exhaustion and the inhibition of the immune response [18]. This is actually the basis for introducing them in the combined positive score (CPS) instead of the tumor proportion score (TPS) in the evaluation of several cancers [19]. Our findings also reinforce the notion of inhibiting this pathway in chordomas. We also found that PD-L1 expression was associated with larger tumors and higher vascularity; vascular density was also associated with higher inflammatory cell presence. These findings probably reveal the pathophysiology of more aggressive tumors: chordomas that manage to reach larger sizes activate an inhibitory phenotype in their immune microenvironment.

Our findings probably contradict those of Zou et al., who showed a better prognosis for more PD-L1-positive stromal cells [14] and a better prognosis for tumors highly infiltrated by CD3, CD8, and CD4 cells [11]. In a large series of soft tissue sarcomas, not including chordomas, T cells were similarly not found to be a prognostic factor [4]. Sarcomas largely inflamed by several immune cell types, including B cells and tertiary lymphoid structures with high endothelial venules, had a better prognosis, but further analysis showed that it was the B cell, and not the T cell, population that was the dominant factor impacting survival [4]. We did not find an association between survival and high B cell expression or high endothelial venules, but both factors were rather uncommon in chordomas, probably reflecting their limited role in these bone sarcomas. In Ewing’s sarcoma family of tumors [20] and in osteosarcomas [21], CD8 was not found to be a prognostic factor either. Similarly, another study of osteosarcomas did not show prognostic significance for CD8 infiltration in the whole cohort, but it was found to be a positive prognostic factor for patients treated with an osteoclast inhibitor [22]. In other cancer types, CD8 T cells can be a positive [2] or negative prognostic factor [23]. These results probably highlight the complexity of the immune microenvironment and suggest that it should be studied as a complex ecosystem, in a tumor- and context-dependent manner, where more than one type of molecule and cell intervenes. We also found a trend for highly vascularized tumors to be associated with worse prognosis, which is in line with the results of Zou et al. [14], and this further substantiates the recent and promising results of patients treated with apatinib, a small-molecule tyrosine kinase inhibitor that selectively binds to VEGFR2, decreasing tumor vascular density [24].

## 5. Conclusions

Our study suffers from limitations associated with its retrospective design, such as those attributed to the aforementioned immunohistochemical techniques. However, retrospective studies are often the only means to expand our knowledge of this rare disease, which, given its nature, requires a very long follow up. Similarly, despite being a large series for such a rare disease, this remains a small series regarding the strengths of the statistical analysis in the various subgroups.

To conclude, in the current chordoma series, we found no tumor cell PD-L1 expression, but we did find a negative prognostic role for PD-L1+ immune cells, supporting the need for further studies in this field and the notion of a possible beneficial role of immunotherapy in these patients.

## Figures and Tables

**Table 1:** Demographics.

Parameter	*n*, %
Age (*n* = 81)	
Range	12–82
Median	64
Mean ± SD	58 ± 17.4
Sex (*n* = 81)	
Female	34, 42%
Male	47, 58%
Localization (*n* = 81)	
Skull	30, 37%
Sacrum	27, 33.3%
Mobile spine	24, 29.7%
Recurrence (*n* = 81)	
Yes	52, 64.2%
No	29, 35.8%
Multiple recurrences (*n* = 80)	
Yes	24, 30%
No	56, 70%
Tumor size (in mm **)	
Range	19–144
Median	43
Mean ± SD	50.6 ± 27.5
Principal treatment at diagnosis (*n* = 85)	
Surgical	72, 84.7%
Radiotherapy	10, 11.8%
Palliative	3, 3.5%
Adjuvant therapy (*n* = 40)	
Radiotherapy	36, 90%
Surgical	3, 7.5%
immunotherapy	1, 2.5%
Follow-up (*n* = 81)	
Range	2–264
Median	60
Mean ± SD	69.5 ± 59.5
Patient status (*n* = 81)	
Alive	66, 81.5%
Dead *	15, 18.5%
Overall survival (*n* = 81)	
Range	2–264
Median (log-rank)	Not reached

* = only disease-associated deaths. *n* denotes the number of cases with available data. ** = size was available for only 38 cases, while tumor volume was not available.

**Table 2:** Immunohistochemical findings.

Parameter	*n*, %
CD20 (*n* = 73)	
Low (score 0–1)	65, 89%
High (score 2–3)	8, 11%
CD8 (*n* = 81)	
Low (score 0–1)	55, 67.9%
High (score 2–3)	26, 32.1%
CD4 (*n* = 41)	
Low (score 0–1)	25, 61%
High (score 2–3)	16, 39%
CD163 (*n* = 74)	
Low (score 0–1)	45, 60.8%
High (score 2–3)	29, 39.2%
PD-L1 inflammatory cells (*n* = 81)	
No (score 0)	60, 74%
Yes (score 1–3)	21, 26%
CD34 vascular density (*n* = 74)	
Range	1–22
Median (High and Low, *n*,%)	3.5 (37, 50% and 37, 50%)
Mean ± SD	5.8 ± 5.7
MECA-79 vessels (*n* = 59)	
High	5, 8.5%
Low	54, 91.5%

**Table 3:** Immunohistochemical comparisons.

Parameter	CD8			CD163			MECA-79			CD34			PD-L1 Inflammatory Cells		
	High	Low	*p*	High	Low	*p*	High	Low	*p*	High	Low	*p*	Yes	No	*p*
CD20															
High	6 (8.3%)	2 (2.7%)	0.01 *	3 (4.1%)	5 (6.9%)	0.8	2 (3.4%)	6 (10.2%)	0.07	4 (5.5%)	4 (5.5%)	0.9	2 (2.7%)	6 (8.2%)	0.9
Low	20 (27.4%)	45 (61.6%)		26 (35.6%)	39 (53.4%)		3 (5.1%)	48 (81.3%)		33 (45.2%)	32 (43.8%)		17 (23.3%)	48 (65.8%)	
CD8															
High				14 (18.9%)	12 (16.2%)	0.05	2 (3.4%)	24 (40.7%)	0.8	22 (29.7%)	4 (5.4%)	<0.0001 ^§^	13 (16.1%)	13 (16.1%)	0.0007 ^£^
Low				15 (20.3%)	33 (44.6%)		3 (5.1%)	30 (50.8%)		15 (20.3%)	33 (44.6%)		8 (9.9%)	47 (58%)	
CD163															
High							2 (3.4%)	26 (44.1%)	0.7	19 (25.7%)	10 (13.5%)	0.03 ^&^	14 (18.9%)	15 (20.2%)	0.0004 °
Low							3 (5.1%)	28 (47.4%)		18 (24.3%)	27 (36.5%)		5 (6.7%)	40 (54%)	
MECA-79															
High										3 (5.1%)	2 (3.4%)	0.8	2 (3.4%)	3 (5.1%)	0.6
Low										30 (50.8%)	24 (40.7%)		16 (27.1%)	38 (64.4%)	
CD34															
High													16 (21.6%)	21 (28.4%)	0.0005 ^+^
Low													3 (4%)	34 (46%)	

Fisher’s exact test *p* values: * 0.02, ^§^ <0.0001, ^£^ 0.001, ^&^ 0.05, ° 0.0007, ^+^ 0.0006. High = scores 2–3 and No = scores 0–1 for CD8, CD163, and CD20. Yes = presence, No = absence for PD-L1. High/Low corresponds to the median as the cut off for CD34 and MECA-79.

**Table 4:** Kaplan–Meier survival analysis.

Parameter	Ten-Year Overall Survival Probability	*p*
CD20		
Low	70%	0.7
High	81%	
CD8		
Low	81%	0.03
High	51%	
CD4		
Low	71%	0.9
High	81%	
CD163		
Low	71%	0.9
High	71%	
PD-L1 inflammatory cells		
No	81%	0.02
Yes	46%	
MECA-79		
High	76%	0.8
Low	61%	
CD34		
Low	81%	0.05
High	60%	
Tumor size		
Low	100%	0.2
High	60%	
Patient age		
Low	82%	0.02
High	58%	
Tissue age		
Low	71%	0.9
High	71%	

Bold denotes statistical significance.

**Table 5:** Multivariate Cox proportional hazards regression analysis.

Variable	HR	CI	*p*
Age	1.031	0.988–1.076	0.1646
CD8 (absence)	0.427	0.106–1.726	0.2325
PD-L1 immune cells (absence)	0.188	0.044–0.815	0.0255

## Data Availability

Data are available upon reasonable request.

## References

[1] Suzuki K., Kachala S.S., Kadota K., Shen R., Mo Q., Beer D.G., Rusch V.W., Travis W.D., Adusumilli P.S. (2011). Prognostic Immune Markers in Non–Small Cell Lung Cancer. Clin. Cancer Res..

[2] Karpathiou G., Casteillo F., Giroult J.-B., Forest F., Fournel P., Monaya A., Froudarakis M., Dumollard J.M., Prades J.M., Peoc’h M. (2017). Prognostic impact of immune microenvironment in laryngeal and pharyngeal squamous cell carcinoma: Immune cell subtypes, immuno-suppressive pathways and clinicopathologic characteristics. Oncotarget.

[3] Camy F., Karpathiou G., Dumollard J.M., Magne N., Perrot J.L., Vassal F., Picot T., Mobarki M., Forest F., Casteillo F. (2020). Brain metastasis PD-L1 and CD8 expression is dependent on primary tumor type and its PD-L1 and CD8 status. J. Immunother. Cancer.

[4] Petitprez F., de Reyniès A., Keung E.Z., Chen T.W.-W., Sun C.-M., Calderaro J., Jeng Y.-M., Hsiao L.-P., Lacroix L., Bougoüin A. (2020). B cells are associated with survival and immunotherapy response in sarcoma. Nature.

[5] Walcott B.P., Nahed B.V., Mohyeldin A., Coumans J.-V., Kahle K.T., Ferreira M.J. (2012). Chordoma: Current concepts, management, and future directions. Lancet Oncol..

[6] Klemen N.D., Kelly C.M., Bartlett E.K. (2021). The emerging role of immunotherapy for the treatment of sarcoma. J. Surg. Oncol..

[7] Migliorini D., Mach N., Aguiar D., Vernet R., Landis B.N., Becker M., McKee T., Dutoit V., Dietrich P.-Y. (2017). First report of clinical responses to immunotherapy in 3 relapsing cases of chordoma after failure of standard therapies. Oncoimmunology.

[8] Mathios D., Ruzevick J., Jackson C.M., Xu H., Shah S., Taube J.M., Burger P.C., McCarthy E.F., Quinones-Hinojosa A., Pardoll D.M. (2015). PD-1, PD-L1, PD-L2 expression in the chordoma microenvironment. J. Neurooncol..

[9] Feng Y., Shen J., Gao Y., Liao Y., Cote G., Choy E., Chebib I., Mankin H., Hornicek F., Duan Z. (2015). Expression of programmed cell death ligand 1 (PD-L1) and prevalence of tumor-infiltrating lymphocytes (TILs) in chordoma. Oncotarget.

[10] Zou M.-X., Guo K.-M., Lv G.-H., Huang W., Li J., Wang X.-B., Jiang Y., She X.-L. (2018). Clinicopathologic implications of CD8+/Foxp3+ ratio and miR-574-3p/PD-L1 axis in spinal chordoma patients. Cancer Immunol. Immunother..

[11] Zou M.-X., Lv G.-H., Wang X.-B., Huang W., Li J., Jiang Y., She X.-L. (2019). Clinical Impact of the Immune Microenvironment in Spinal Chordoma: Immunoscore as an Independent Favorable Prognostic Factor. Neurosurgery.

[12] Zou M., Pan Y., Huang W., Zhang T., Escobar D., Wang X., Jiang Y., She X., Lv G., Li J. (2020). A four-factor immune risk score signature predicts the clinical outcome of patients with spinal chordoma. Clin. Transl. Med..

[13] Zou M.-X., Peng A.-B., Lv G.-H., Wang X.-B., Li J., She X.-L., Jiang Y. (2016). Expression of programmed death-1 ligand (PD-L1) in tumor-infiltrating lymphocytes is associated with favorable spinal chordoma prognosis. Am. J. Transl. Res..

[14] Zou M.-X., Zheng B.-W., Liu F.-S., Wang X.-B., Hu J.-R., Huang W., Dai Z.-H., Zhang Q.-S., Liu F.-B., Zhong H. (2019). The Relationship Between Tumor-Stroma Ratio, the Immune Microenvironment, and Survival in Patients With Spinal Chordoma. Neurosurgery.

[15] Ruscetti M., Morris J.P., Mezzadra R., Russell J., Leibold J., Romesser P.B., Simon J., Kulick A., Ho Y., Fennell M. (2020). Senescence-Induced Vascular Remodeling Creates Therapeutic Vulnerabilities in Pancreas Cancer. Cell.

[16] Torlakovic E., Lim H.J., Adam J., Barnes P., Bigras G., Chan A.W.H., Cheung C.C., Chung J.-H., Couture C., Fiset P.O. (2020). “Interchangeability” of PD-L1 immunohistochemistry assays: A meta-analysis of diagnostic accuracy. Mod. Pathol..

[17] Boothman A.-M., Scott M., Ratcliffe M., Whiteley J., Dennis P.A., Wadsworth C., Sharpe A., Rizvi N.A., Garassino M.C., Walker J. (2019). Impact of Patient Characteristics, Prior Therapy, and Sample Type on Tumor Cell Programmed Cell Death Ligand 1 Expression in Patients with Advanced NSCLC Screened for the ATLANTIC Study. J. Thorac. Oncol..

[18] Paver E.C., Cooper W.A., Colebatch A.J., Ferguson P.M., Hill S.K., Lum T., Shin J.-S., O’Toole S., Anderson L., Scolyer R.A. (2021). Programmed death ligand-1 (PD-L1) as a predictive marker for immunotherapy in solid tumours: A guide to immunohistochemistry implementation and interpretation. Pathology.

[19] Emancipator K., Huang L., Aurora-Garg D., Bal T., Cohen E.E.W., Harrington K., Soulières D., Le Tourneau C., Licitra L., Burtness B. (2021). Comparing programmed death ligand 1 scores for predicting pembrolizumab efficacy in head and neck cancer. Mod. Pathol..

[20] Machado I., López-Guerrero J.A., Scotlandi K., Picci P., Llombart-Bosch A. (2018). Immunohistochemical analysis and prognostic significance of PD-L1, PD-1, and CD8+ tumor-infiltrating lymphocytes in Ewing’s sarcoma family of tumors (ESFT). Virchows Arch..

[21] Zhang C., Zheng J.-H., Lin Z.-H., Lv H.-Y., Ye Z.-M., Chen Y.-P., Zhang X.-Y. (2020). Profiles of immune cell infiltration and immune-related genes in the tumor microenvironment of osteosarcoma. Aging.

[22] Gomez-Brouchet A., Illac C., Gilhodes J., Bouvier C., Aubert S., Guinebretiere J.-M., Marie B., Larousserie F., Entz-Werlé N., de Pinieux G. (2017). CD163-positive tumor-associated macrophages and CD8-positive cytotoxic lymphocytes are powerful diagnostic markers for the therapeutic stratification of osteosarcoma patients: An immunohistochemical analysis of the biopsies fromthe French OS2006 phase 3 t. Oncoimmunology.

[23] Fusco N., Vaira V., Righi I., Sajjadi E., Venetis K., Lopez G., Cattaneo M., Castellani M., Rosso L., Nosotti M. (2020). Characterization of the immune microenvironment in malignant pleural mesothelioma reveals prognostic subgroups of patients. Lung Cancer.

[24] Liu C., Jia Q., Wei H., Yang X., Liu T., Zhao J., Ling Y., Wang C., Yu H., Li Z. (2020). Apatinib in patients with advanced chordoma: A single-arm, single-centre, phase 2 study. Lancet Oncol..

